# Concurrent Validity of the Foot Health Status Questionnaire and Study Short Form 36 for Measuring the Health-Related Quality of Life in Patients with Foot Problems

**DOI:** 10.3390/medicina55110750

**Published:** 2019-11-19

**Authors:** Patricia Palomo-López, Daniel López-López, Ricardo Becerro-de-Bengoa-Vallejo, Marta Elena Losa-Iglesias, David Rodríguez-Sanz, Josué Fernández-Carnero, João Martiniano, César Calvo-Lobo

**Affiliations:** 1 University Center of Plasencia, Universidad de Extremadura, 10600 Plasencia, Spain; patibiom@unex.es; 2 Research, Health and Podiatry Group, Department of Health Sciences, Faculty of Nursing and Podiatry, Universidade da Coruña, 15403 Ferrol, Spain; 3 School of Nursing, Physiotherapy and Podiatry, Universidad Complutense de Madrid, 28040 Madrid, Spain; ribebeva@ucm.es (R.B.-d.-B.-V.); davidrodriguezsanz@ucm.es (D.R.-S.); cescalvo@ucm.es (C.C.-L.); 4 Faculty of Health Sciences, Universidad Rey Juan Carlos, 28922 Alcorcón, Spain; marta.losa@urjc.es; 5 Department of Physical Therapy, Occupational Therapy, Rehabilitation and Physical Medicine, Rey Juan Carlos University, La Paz Hospital Institute for Health Research, IdiPAZ, Grupo Multidisciplinar de Investigación y Tratamiento del Dolor, Grupo de Excelencia Investigadora URJC-Banco de Santander, 28922 Madrid, Spain; josue.fernandez@urjc.es; 6 Escola Superior de Saúde da Cruz Vermelha Portuguesa, 1300-125 Lisboa, Portugal; jmartiniano@esscvp.eu

**Keywords:** foot, foot deformities, foot diseases, musculoskeletal diseases, quality of life

## Abstract

*Background and Objectives:* Foot problems may be considered to be a prevalent condition and impact the health-related quality of life (QoL). Considering these Spanish-validated tools, the Foot Health Status questionnaire (FHSQ) may provide a health-related QoL measurement for specific foot conditions and general status. To date, the domains of the FHSQ and Medical Outcomes Study Short Form 36 (SF-36) have not been correlated. Therefore, the main aim of this study was to correlate the domains of the FHSQ and SF-36 in patients with foot problems. *Materials and Methods:* A cross-sectional descriptive study was carried out. A sample of 101 patients with foot problems was recruited. A single researcher collected descriptive data, and outcome measurements (FHSQ and SF-36) were self-reported. *Results:* Spearman’s correlation coefficients (*r*_s_) were calculated and categorized as weak (*r*_s_ = 0.00–0.40), moderate (*r*_s_ = 0.41–0.69), or strong (*r*_s_ = 0.70–1.00). In all analyses, statistical significance was considered with a *p*-value < 0.01 with a 99% confidence interval. Statistically significant differences (*p* < 0.01) were found between all domains of FHSQ and SF-36, except for the mental health domain of the SF-36 with foot pain, foot function, and general foot health of the FHSQ, as well as between the vitality domain of the SF-36 and the general foot health domain of the FHSQ (*p* > 0.01). Statistically significant correlations varied from week to strong (*r*_s_ = 0.25–0.97). The strongest correlations (*p* < 0.001) were found for physical activity and physical function (*r*_s_ = 0.94), vigor and vitality (*r*_s_ = 0.89), social capacity and social function (*r*_s_ = 0.97), and general health domains of the SF-36 and FHSQ. *Conclusions:* The FHSQ and SF-36 showed an adequate concurrent validity, especially for the physical activity or function, vigor or vitality, social capacity or function, and general health domains. Nevertheless, the mental health domain of the SF-36 should be considered with caution.

## 1. Introduction

Foot problems are common disorders, which have reached prevalence rates from 61% to 79% [1,2,3]. Nevertheless, further studies about the burden of foot problems should be carried out, in order to state if these conditions comprise a major public health problem [4,5,6]. Foot problems may frequently be chronic conditions that appear at primary care consultations, and can reduce the health-related quality of life (QoL), as well as balance and gait, and increase the risk of falls. Furthermore, foot problems may be more prevalent and foot surgery costs may be higher in females and older adults [7,8,9].

Foot problems are frequently complex conditions that affect individuals differently, depending on presentation, structures involved, and characteristics of symptoms [10]. Up to 24% of people have diagnosed foot and ankle pathologies, most commonly observed in the forefoot [8].

Foot health-related QoL impairments have been shown in patients with foot problems regarding specific foot structures, such as hallux valgus [11,12], plantar fasciitis [10,13], or lesser toe deformities [14], as well as systemic diseases, such as breast cancer [15], diabetes [16], or rheumatoid arthritis [17].

Multiple Spanish-validated and reliable tools, such as the Foot Health Status Questionnaire (FHSQ) [10,18,19,20,21], Foot Function Index (FFI) [22,23,24], Manchester Foot Pain and Disability Index (MFPDI) [25], and Bristol Foot Score (BFS) [26,27], have been used to evaluate specific foot health-related QoL issues. Of the available tools, the FHSQ has been preferred, due to its high validity and an included measure of general health [10]. Furthermore, the FHSQ provided a domain for measuring general health, while the other questionnaires did not [10,18,19,20,21].

Considering general health-related QoL, the Medical Outcomes Study Short Form 36 (SF-36) may be considered as a widely used generic and Spanish-validated scale for evaluating the general health status of patients [28,29]. Almost four decades ago, health-related QoL tools were developed to specify patients’ everyday needs, wellbeing, and problems, as well as determine their physical and psychological status [30].

Considering these Spanish-validated tools, the FHSQ may provide a health-related QoL measurement for specific foot conditions and general status [10,18,19,20,21]. To date, the domains of the FHSQ and SF-36 have not been correlated [28,29]. Therefore, the main aim of this study was to correlate the domains of the FHSQ and SF-36 in patients with foot problems. Furthermore, the second purpose was to evaluate the score differences of the FHSQ and SF-36 between males and females in our sample.

## 2. Materials and Methods

### 2.1. Design

A cross-sectional descriptive study was carried out, in order to correlate the Spanish versions of the FHSQ [21] and SF-36 [29] in patients with foot problems following the “Strengthening the Reporting of Observational Studies in Epidemiology” guidelines and checklist [31]. This research was approved the Ethics Committee of the University of Extremadura (Spain) (number 25/2018, approved on 6 March 2018). Before the start of the study, all patients voluntarily signed their consent inform form. Furthermore, the Helsinki Declaration, human rights, and biomedicine statements for the human research ethical standards were considered.

### 2.2. Sample

A total sample of 101 patients with foot problems was recruited by a consecutive sampling method at the Podiatric Medicine and Surgery Clinic for the treatment of foot problems at the University of Extremadura (Plasencia, Spain) from July to December 2017. The age range varied from 21 to 89 years old, according to the questionnaire recommendations [28]. The exclusion criteria included prior history in the patient’s medical record of pathological fractures, trauma, surgery, active systemic neoplasia, infection, neurologic conditions, and lack of autonomy in daily life activities, as well as refusal to sign the informed consent form, inability to understand the instructions to perform the present study, and nationalities other than Spanish [11].

### 2.3. Procedure

After applying the inclusion and exclusion criteria, a single trained researcher collected the data and outcome measurements at the same day. First of all, sociodemographic and descriptive data were registered. Second, the order of registering the outcome measurements (FHSQ and SF-36) was randomized by opaque and closed envelopes, in order to determine the first and second questionnaire provide to each patient. Both questionnaires were administered in person at the Podiatric Medicine and Surgery Clinic for the treatment of foot problems at the University of Extremadura (Plasencia, Spain), and were self-reported by each patient.

### 2.4. Sociodemographic and Descriptive Data

The sociodemographic data included age, sex, weight, height, body mass index (calculated using Quetelet’s equation: BMI = kg/m²) [32], professional activity (student, freelance, employed worker, unemployed, or retired), study level (incomplete primary, complete primary, secondary, degree, or superior degree) and civil status (single, divorced, widowed, couple, or married). In addition, the foot side (left, right, or both) and foot problem region (forefoot, midfoot, hindfoot, or several regions) were recorded. Finally, the type of foot problems (hallux valgus or bunions; metatarsalgia; plantar heel pain, plantar fasciitis or heel spurs; onychocryptosis, toe deformities; Morton’s neuroma; pes cavus; pes planus; warts; helomas; hyperkeratosis; and generalized foot pain), predisposing factors (diabetes, obesity, depression, vascular disease, or osteoarticular pathology), and sport participation were collected.

### 2.5. FHSQ Outcome Measurement

This Spanish-validated, self-administered tool (FHSQ, 1.03 Version) was comprised of three sections. Section 1 was composed of 13 items and was divided into four specific, foot health-related domains: foot function (four items), foot pain (four items), footwear (three items), and general foot health (two items). A high degree, considering content, criterion, and construct validity (Cronbach α = 0.89–0.95), as well as high retest intraclass correlation coefficient reliability (ICC = 0.74–0.92), was shown for this section. Section 2 was comprised of four domains reflecting overall health: general health, physical activity, social capacity, and vigor, whose items were initially adapted from the SF-36. Lastly, Section 3 was comprised of information about socioeconomic status, comorbidity, and satisfaction, as well as medical record data, which were included in the descriptive data. Each item provided several options by a Likert-type ordinal scale, with only one response item as the most appropriate. The questionnaire provided a score for each domain, which was obtained by a computer program (score ranged from 0 to 100). Regarding the health-related QoL, the 0 score reflected the worst status, and the 100 score showed the best status [10,18,19,20,21].

### 2.6. SF-36 Outcome Measurement

The SF-36 (SF-36v2 Standard, Spanish 2.0 Version) was composed of eight health-related domains, such as physical function, physical role, mental health, vitality, emotional role, social function, body pain, and general health. This Spanish-validated and reliable tool showed an adequate Cronbach’s Alpha (Cronbach α = 0.71–0.94), except for the social function scale (Cronbach α = 0.45) and intraclass correlation coefficient (ICC = 0.58–0.99). Also, each domain presented a score from 0 to 100; the 0 score reflected the worst QoL, and the 100 score the best health-related QoL [28,29].

### 2.7. Sample Size Calculation

A sample size was calculated using the point biserial model correlation with the G*Power 3.1.9.2 software (Heinrich-Heine-Universität Düsseldorf; Düsseldorf, Germany). Indeed, a two-tailed hypothesis, a moderate effect size of 0.4, an α error probability of 0.01, and a power (1-β error probability) of 0.95 were used for the sample size calculation. Therefore, a total sample size of 97 subjects was calculated. Finally, a total sample size of 101 patients was included in this study.

### 2.8. Statistical Analysis

The Kolmogorov–Smirnov test was utilized to assess normality, and data were determined as normally distributed if *p* > 0.05. Considering the quantitative data, the normality test results indicated that all data were not normally distributed, except for weight and BMI of the demographic data. Non-parametric data, such as median, interquartile range (IR), and minimum–maximum (range), were used to describe all data except for the parametric data (weight and BMI), which were described as mean, standard deviation (SD), and minimum–maximum (range). Regarding the categorical variables (sex, professional activity, study level, civil status, side, and region of foot problems), frequency and percentages were applied to describe the data.

With respect to the comparison of quantitative data between males and females for both tests domains (FHSQ and SF-36), independent Student *t*-tests were performed to determine whether statistically significant differences were obtained for the parametric data (weight and BMI), while Mann–Whitney U tests were applied for the non-parametric data (rest of variables). For the categorical variables, a Chi-squared test was utilized to determine whether a significant difference was found between the observed frequencies.

In order to determine the non-parametric correlations between the eight domains (foot function, foot pain, footwear, general foot health, general health, physical activity, social capacity, and vigor) of the FHSQ [10,18,19,20,21] and the eight domains (physical function, physical role, mental health, vitality, emotional role, social function, body pain, and general health) of the SF-36, Spearman’s correlation coefficients (*r*_s_) were calculated and categorized as weak (*r*_s_ = 0.00–0.40), moderate (*r*_s_ = 0.41–0.69), or strong (*r*_s_ = 0.70–1.00) [33,34].

In all analyses, statistical significance was considered with a *p*-value < 0.01 with a 99% confidence interval (CI). All analyses were carried out with the available statistical software SPSS version 22.0 for Windows (SPSS Inc., Chicago, IL, United States).

## 3. Results

### 3.1. Sociodemographic and Descriptive Data

A total sample of 101 patients with foot problems, with an age range of 21 to 89 years old, completed the research. The sample included 83 (82.17%) females and 18 (17.82%) males. Table 1 shows the demographic and descriptive data of the sample. Despite the fact that the majority of the patients were overweight (BMI = 27.51 ± 4.82 kg/m^2^), the only statistically significant differences (*p* < 0.01) between males and females were shown for height and weight, and not for age, BMI, foot problem (FP) region, or side (*p* > 0.01).

Table 2 shows the social characteristics of the sample. There were not any statistically significant differences (*p* > 0.01) for the studied social characteristics, such as professional activity, study level, and civil status.

Regarding the descriptive data for the foot problems, the foot problem percentages (frequency) presented by the patients of the sample were 15.84% (*n* = 16) hallux valgus or bunions; 20.79% (*n* = 21) metatarsalgia; 13.86% (*n* = 14) plantar heel pain, plantar fasciitis, or heel spurs; 11.88% (*n* = 12) onychocryptosis; 10.89% (*n* = 11) toe deformities; 0.99 % (*n* = 1) Morton’s neuroma; 9.90% (*n* = 10) pes cavus; 2.87% (*n* = 3) pes planus; 1.98% (*n* = 2) warts; 4.95% (*n* = 5) helomas; and 4.95% (*n* = 5) hyperkeratosis; as well as (*n* = 17) generalized foot pain. In addition, the percentages (frequency) of patients with foot problems who presented predisposing factors were 21.78 % (*n* = 22) vascular disease, 14.85 % (*n* = 15) osteoarticular pathology, 8.91% (*n* = 9) diabetes, 2.97% (*n* = 3) obesity, and 1.98% (*n* = 2) depression. Furthermore, 0.69% (*n* = 7) of these patients reported sport participation.

### 3.2. FHSQ by Sex Distribution

The FHSQ scores between males and females with foot problems are shown in Table 3. Females with foot problems showed statistically significant differences (*p* < 0.01) for lower scores in the domains of foot pain and general health. The rest of domains did not show statistically significant differences (*p* > 0.01).

### 3.3. SF-36 by Sex Distribution

The SF-36 scores between males and females with foot problems are shown in Table 4. Females with foot problems showed statistically significant differences (*p* < 0.01) for lower scores in the domain of general health. The rest of the domains did not show statistically significant differences (*p* > 0.01).

### 3.4. Correlations Between FHSQ and SF-36 Domains

The Spearman’s correlations between FHSQ and SF-36 domains are shown in Table 5. Statistically significant differences (*p* < 0.01) were found between all domains of the FHSQ and SF-36, except for the mental health domain of the SF-36 with the foot pain, foot function, and general foot health of the FHSQ, as well as between the vitality domain of the SF-36 and the general foot health domain of the FHSQ (*p* > 0.01). Statistically significant correlations varied from weak to strong (*r*_s_ = 0.25–0.97). The strongest correlations (*p* < 0.001) were found for physical activity and physical function (*r*_s_ = 0.94), vigor and vitality (*r*_s_ = 0.89), social capacity and social function (*r*_s_ = 0.97), and the general health domains of the SF-36 and FHSQ.

## 4. Discussion

To the authors’ knowledge, this research may be considered as the first study to show the concurrent validity between the FHSQ [10,18,19,20,21] and SF-36 [28,29] domains in patients with foot problems. Gender differences were in line with a prior study of ours, which assessed FHSQ scores in patients with foot problems [35], and these differences were in accordance with our findings using the SF-36. The correlation between both questionnaires provides the concurrent validity of each domain to the current research literature, and supports the use of the FHSQ as an adequate tool to measure QoL in relation to general health and in conjunction with specific foot health, using only a questionnaire. This issue may be considered as an advantage with respect to the other available Spanish-validated questionnaires to evaluate specific foot-health-related QoL, such as the FFI [22,23,24], MFPDI [25] and BFS [26,27].

The high prevalence (72.1%) of foot problems in the population requires health-related QoL research. According to our study, foot pain tends to be more prevalent in females than in males [30,36]. In line with our research, prior studies have shown that QoL related to foot health presents lower scores, showing a worse QoL in females than in males [37]. Furthermore, specific foot problems, such as hallux valgus, calcaneal apophysitis, foot arch height, heel pain, or onychomycosis have been shown to reduce the QoL related to foot health, and these results coincide with our studies [13,38,39,40,41]. Thus, foot problems impair the QoL related to foot and general health—and specifically, women with foot problems present a negative QoL impact related to foot and general health with respect to men, except in the overall health and social capacity domains, which appear to be linked to the presence of foot conditions and the aging process [35,42].

According to the concurrent validity, the mental health domain of the SF-36 showed the worst correlations. This fact may be due to the fact that the FHSQ did not include any specific domain for mental health. Nevertheless, the strongest correlations were determined for physical activity or function, vigor or vitality, social capacity or function, and general health domains. The possible reason for this may be due initially to Section 2 of the FHSQ, which was based on the domains of the SF-36. Nevertheless, this is the first study to determine their concurrent validity [10,18,19,20,21,28,29].

Future studies should address the need to use and study scores that are not limited just to the functionality of the foot. Despite the fact that the SF-36 has been widely used to determine general quality of life related to foot disorders [43,44,45], the short form (SF)-12 score has also been used to compare general health-related QoL outcomes after foot and ankle interventions [46,47,48]. Nevertheless, we used the SF-36, since Section 2 of the FHSQ was initially adapted from the SF-36, and this study was necessary in order to correlate both domains [10,18,19,20,21].

Several limitations of this study should be acknowledged. A sample of diverse patients from other countries could be helpful to improve the strength of this study. This study only evaluated the concurrent validity between FHSQ and SF-36 in Spanish patients with foot problems. Although foot problems are very common in the population [49], this study should be expanded to other regions with more frequent musculoskeletal problems, such as the lower back, neck, or shoulder regions [50]. In addition, the used consecutive sampling method may present a bias, and a randomized sampling should be used in future studies. According to our prior affirmations, we used the SF-36, but the SF-12 should be correlated with the FHSQ in futures studies, because SF-12 may be considered a reliable and summarized questionnaire of the SF-36, and has been widely used to evaluate the health-related QoL of patients with foot and ankle disorders [46,47,48]. Finally, the impact of the correlation between the different foot pathologies, including different congenital and acquired or traumatic disorders and degenerative pathologies, was not analyzed in the present study, because our sample was not balanced enough to carry out these comparisons. The authors encourage researchers to perform future studies addressing health-related QoL impacts under these different conditions.

## 5. Conclusions

The FHSQ and SF-36 showed an adequate concurrent validity, especially for physical activity or function, vigor or vitality, social capacity or function, and general health domains. Nevertheless, the mental health domain of the SF-36 should be considered with caution.

## Figures and Tables

**Table 1:** Demographic and descriptive data of the sample.

Demographic and Descriptive Data		Total Group *n* = 101	Male *n* = 18	Female *n* = 83	*p*-Value
Age (years)		64.00 ± 23.50 (21–89)	59.50 ± 26.25 (21–89)	65.00 ± 22.00 (21–87)	0.607 ^†^
Weight (kg)		72.46 ± 13.114 (33–107)	81.11 ± 14.04 (59–104)	70.59 ± 12.72 (33–107)	0.002 *
Height (cm)		161.00 ± 10.00 (148–187)	170.00 ± 10.00 (162–187)	160.00 ± 9.00 (148–174)	<0.001 ^†^
BMI (kg/m^2^)		27.51 ± 4.824 (13.22–38.83)	27.20 ± 3.9 (21.94–36.06)	27.58 ± 5.00 (13.22–38.83)	0.765 *
FP region	Forefoot	67 (66.3%)	13 (72.2%)	54 (65.0%)	0.498 ^‡^
	Midfoot	2 (2.0%)	1 (5.5%)	1 (1.2%)	
	Hindfoot	12 (11.9%)	1 (5.5%)	11 (13.2%)	
	Several	20 (19.8%)	3 (16.6%)	17 (20.4%)	
FP side	Left	14 (13.9%)	4 (22.2%)	10 (12.0%)	0.483 ^‡^
	Right	16 (15.8%)	2 (11.1%)	14 (12.86%)	
	both sides	71 (70.3%)	12 (66.6%)	59 (71.08%)	

BMI: body mass index; FP: foot problem. * Mean ± standard deviation, range (min–max) and Student´s *t*-test for independent samples were applied. ^†^ Median ± interquartile range, range (min–max) and Mann–Whitney U test were used. ^‡^ Frequency, percentage (%), and Chi-squared test (*χ*^2^) were utilized. In all the analyses, *p* < 0.01 (with a 99% confidence interval) was considered statistically significant.

**Table 2:** Social characteristics of the sample.

Social Characteristics		Total Group *n* = 101	Male *n* = 18	Female *n* = 83	*p*-Value ^‡^
Professional activity	Student	4 (4%)	1 (5.5%)	3 (3.6%)	0.270
	Freeland	8 (7.9%)	2 (11.1%)	6 (7.2%)	
	Employed	23 (22.8%)	7 (38.8%)	16 (19.2%)	
	unemployed	7 (6.9%)	0 (0.0%)	7 (8.4%)	
	Retired	59 (58.4%)	8 (44.4%)	51 (61.45)	
Study level	I. primary	28 (27.7%)	1 (5.5%)	27 (32.5%)	0.033
	C. primary	36 (35.6%)	8 (44.4%)	28 (33.7%)	
	Secondary	21 (20.8%)	4 (22.2%)	17 (20.4%)	
	Degree	11 (10.9%)	2 (11.1%)	9 (10.8%)	
	S. degree	5 (5.0%)	3 (16.6%)	2 (2.4)	
Civil status	Single	16 (15.8%)	1 (5.5%)	15 (18.0%)	0.090
	Divorced	3 (3.0%)	1 (5.5%)	2 (2.4%)	
	Widowed	23 (22.8%)	1 (5.5%)	22 (26.5%)	
	Couple	2 (2.0%)	0 (0.0%)	2 (2.4%)	
	Married	57 (56.4%)	15 (83.33%)	42 (50.6%)	

C: complete; I: incomplete; S: superior. ^‡^ Frequency, percentage (%), and Chi-squared test (*χ*^2^) were utilized. In all the analyses, *p* < 0.01 (with a 99% confidence interval) was considered statistically significant.

**Table 3:** Comparisons of FHSQ scores between males and females with foot problems.

FHSQ Domains.	Total group Median ± IR (Range) *n* = 100	Male Median ± IR (Range) *n* = 18	Female Median ± IR (Range) *n* = 83	*p*-value Male vs. Female ^†^
Foot pain	48.12 ± 37.50 (0–90)	63.43 ± 42.66 (25–90)	41.87 ± 41.25 (0–87)	0.009
Foot function	68.75 ± 46.88 (0–100)	78.12 ± 45.31 (25–100)	68.75 ± 50.00 (0–100)	0.562
Footwear	25.00 ± 50.00 (0–100)	25.00 ± 54.17 (0–100)	25.00 ± 50.00 (0–100)	0.674
General foot health	25.00 ± 21.25 (0–85)	25.00 ± 38.13 (0–72)	25.00 ± 12.50 (0–85)	0.063
General health	60.00 ± 45.00 (0–100)	85.00 ± 42.50 (20–100)	60.00 ± 50.00 (0–100)	0.002
Physical activity	72.22 ± 47.22 (55–100)	80.55 ± 27.68 (22–100)	66.66 ± 50.00 (5–100)	0.097
Social capacity	87.50 ± 37.50 (0–100)	93.75 ± 28.13 (37–100)	87.50 ± 37.50 (0–100)	0.346
Vigor	62.50 ± 31.25 (0–100)	68.75 ± 37.50 (12–93)	56.25 ± 37.50 (0–100)	0.087

IR: interquartile range; FHSQ: Foot Health Status Questionnaire. ^†^ Median ± interquartile range, range (min–max) and Mann–Whitney U test were used. In all the analyses, *p* < 0.01 (with a 99% confidence interval) was considered statistically significant.

**Table 4:** Comparisons of SF-36 scores between males and females with foot problems.

SF-36 Domains	Total group Median ± IR (Range) *n* = 100	Male Median ± IR (Range) *n* = 18	Female Median ± IR (Range) *n* = 83	*p*-value Male vs. Female ^†^
Physical function	75.00 ± 42.50 (5–100)	85.00 ± 27.50 (25–100)	65.00 ± 45.00 (5–100)	0.059
Physical role	00.00 ± 00.00 (0–100)	00.00 ± 00.00 (0–100)	00.00 ± 25.00 (0–100)	0.064
Mental health	00.00 ± 00.00 (0–100)	00.00 ± 00.00 (0–66)	00.00 ± 00.00 (0–100)	0.412
Vitality	56.30 ± 31.20 (0–100)	68.80 ± 37.50 (25–93)	56.30 ± 31.20 (0–100)	0.123
Emotional role	65.00 ± 25.00 (20–85)	70.00 ± 21.25 (40–85)	65.00 ± 25.00 (20–85)	0.257
Social function	87.50 ± 42.00 (0–100)	93.75 ± 28.13 (12–100)	87.50 ± 46.00 (0-100)	0.270
Body Pain	37.50 ± 25.00 (0–100)	47.45 ± 45.63 (12–100)	37.50 ± 25.00 (0–100)	0.090
General health	50.00 ± 25.00 (0–100)	80.00 ± 27.50 (35–95)	55.00 ± 35.00 (0–95)	0.001

IR: interquartile range; SF-36: Medical Outcomes Study Short Form 36. ^†^ Median ± interquartile range, range (min–max) and Mann–Whitney U test were used. In all the analyses, *p* < 0.01 (with a 99% confidence interval) was considered statistically significant.

**Table 5:** Spearman´s correlations between FHSQ and SF-36 scores in patients with foot problems.

FHSQ Domains	SF-36 Domains: *r*_s_ (*p*-value) *							
	Physical Function	Physical Role	Mental Health	Vitality	Emotional Role	Social Function	Body Pain	General Health
Foot pain	0.29 (0.003)	−0.36 (<0.001)	−0.13 (0.177)	0.33 (0.001)	0.25 (0.009)	0.30 (0.002)	-0.45 (<0.001)	-0.39 (<0.001)
Foot function	0.52 (<0.001)	−0.40 (<0.001)	−0.21 (0.030)	0.51 (<0.001)	0.42 (<0.001)	0.49 (<0.001)	0.50 (<0.001)	0.49 (<0.001)
Footwear	0.32 (0.001)	−0.28 (0.004)	−0.34 (<0.001)	0.44 (<0.001)	0.27 (0.006)	0.31 (0.001)	0.28 (0.003)	0.33 (0.001)
General foot health	0.33 (0.001)	−0.30 (0.002)	−0.13 (0.174)	0.24 (0.013)	0.32 (0.001)	0.32 (0.001)	0.41 (<0.001)	0.38 (<0.001)
General health	0.69 (<0.001)	−0.51 (<0.001)	−0.33 (0.001)	0.56 (<0.001)	0.59 (<0.001)	0.51 (<0.001)	0.41 (<0.001)	0.91 (<0.001)
Physical activity	0.94 (<0.001)	−0.49 (<0.001)	−0.44 (<0.001)	0.62 (<0.001)	0.56 (<0.001)	0.51 (<0.001)	0.50 (<0.001)	0.58 (<0.001)
Social capacity	0.45 (<0.001)	−0.44 (<0.001)	−0.31 (0.002)	0.55 (<0.001)	0.56 (<0.001)	0.97 (<0.001)	0.44 (<0.001)	0.54 (<0.001)
Vigor	0.61 (<0.001)	−0.56 (<0.001)	−0.37 (<0.001)	0.89 (<0.001)	0.47 (<0.001)	0.57 (<0.001)	0.50 (<0.001)	0.56 (<0.001)

FHSQ: Foot Health Status Questionnaire; *r*_s_: Pearson´s correlations coefficient; SF-36: Medical Outcomes Study Short Form 36. * Pearson´s correlations coefficient (*r*_s_) and *p*-value were applied. In all the analyses, *p* < 0.01 (with a 99% confidence interval) was considered statistically significant.
